# Bivalent mRNA booster vaccination recalls cellular and antibody immunity against antigenically divergent SARS-CoV-2 spike antigens

**DOI:** 10.1038/s41541-025-01129-6

**Published:** 2025-04-18

**Authors:** Mai-Chi Trieu, Arnold Reynaldi, Wen Shi Lee, Hyon-Xhi Tan, Andrew Kelly, Robyn Esterbauer, Rebecca J. Cox, Jennifer Audsley, Joseph Sasadeusz, David S. Khoury, Miles P. Davenport, Deborah Cromer, Adam K. Wheatley, Stephen J. Kent, Jennifer A. Juno

**Affiliations:** 1 Department of Microbiology and Immunology, University of Melbourne, Peter Doherty Institute for Infection and Immunity, Melbourne, Victoria Australia; 2 Influenza Centre, Department of Clinical Science, University of Bergen, Bergen, Norway; 3 Kirby Institute, University of New South Wales, Kensington, New South Wales Australia; 4 Department of Microbiology, Haukeland University Hospital, Bergen, Norway; 5 Department of Infectious Diseases, Peter Doherty Institute for Infection and Immunity, University of Melbourne and Royal Melbourne Hospital, Melbourne, Victoria Australia; 6 Victorian Infectious Diseases Service, Royal Melbourne Hospital, Melbourne, VIC Australia; 7 Melbourne Sexual Health Centre and Department of Infectious Diseases, Alfred Hospital and Central Clinical School, Monash University, Melbourne, Victoria Australia

**Keywords:** RNA vaccines, Viral infection, Adaptive immunity

## Abstract

The ongoing rollout of SARS-CoV-2 vaccines lags behind rapid viral evolution. Updated vaccine immunogens elicit neutralising antibodies against the component strain. However, protection against future SARS-CoV-2 variants is unclear. Here, we sought to understand factors underpinning serological breadth following bivalent BA.1 vaccination. Booster vaccination of 33 individuals elicited robust and durable antibody responses against component vaccine antigens and elevated frequencies of spike-specific CD4 and CD8 T cells. Immunisation predominantly drove recall of cross-reactive memory B cells which also recognised XBB.1.5 spike, with significantly enhanced neutralisation titres against XBB virus seen within 91% of participants. Multivariate regression indicated that both baseline neutralising titres and spike-specific CD4 T cell frequencies were strong predictors of ancestral, BA.1 and XBB neutralisation post-immunisation. These data highlight that updated SARS-CoV-2 vaccines recall cross-reactive memory that maintains recognition of antigenically evolved viral variants and suggests T cell help and prior antibody titres underpin robust vaccine-induced neutralising activity.

## Introduction

The incredibly rapid development and deployment of first-generation vaccines for COVID-19 was a significant technological achievement and served to dramatically reduce the health burden of the SARS-CoV-2 pandemic. However, while relatively stable during the initial phase of global spread, SARS-CoV-2 has since demonstrated a capacity for rapid evolution and antigenic change. Consequently, major losses of vaccine effectiveness against the acquisition of SARS-CoV-2 infection have been observed^1^, and the deployment of updated vaccines targeting variant SARS-CoV-2 strains has lagged behind the continued evolution of the virus.

As such, there is a need to better understand the capacity of repeated immunisation with updated COVID-19 vaccines to maintain protective immunity in the population. Initial bivalent and more recent monovalent boosters for Omicron family variants appear to restore protection against matched circulating viruses^2,3^. Nonetheless, the effectiveness of booster vaccines for eliciting broadly protective antibody responses to cover future viruses is unclear, as are the variables that most strongly impact post-booster antibody titres (which, for example, may include baseline immune responses, time since last antigen exposure, history of infection/vaccination with ancestral or omicron variants, or other demographic factors).

The first COVID-19 vaccines encoding an updated spike approved for use in Australia were BA.1/ancestral bivalent mRNA boosters that became available in late 2022. Here we sought to understand how immune history and time since the last antigen encounter can impact the immune response to both the vaccine antigens and future circulating viral variants. We recruited participants receiving the Moderna mRNA-1273.214 COVID-19 booster vaccine (BA.1/ancestral bivalent mRNA), evaluated both humoral and cellular immune responses to the vaccine antigens and assessed cross-reactivity to XBB/XBB.1.5 subvariants that became predominant during the study in early 2023. We found that the bivalent vaccine boosted neutralising antibodies to high levels against both ancestral and Omicron BA.1 viruses, with cross-reactivity to Omicron XBB. Interestingly, multivariate modelling demonstrated that the strongest predictors of post-booster XBB neutralising titres were a combination of baseline spike-specific CD4 T cell frequencies and pre-existing neutralising antibody titres. Our findings suggest that vaccine-induced neutralising antibodies were most likely generated from memory responses, although low levels of de novo BA.1-specific memory B cell responses were elicited upon vaccination. This study provides insights into the collaboration of T and B-cell responses after bivalent ancestral/Omicron BA.1-containing COVID-19 booster vaccination and emphasises the role of memory CD4 T cells and baseline antibody titres in influencing antibody breadth against new viral variants.

## Results

### Study design

**Fig. 1:**
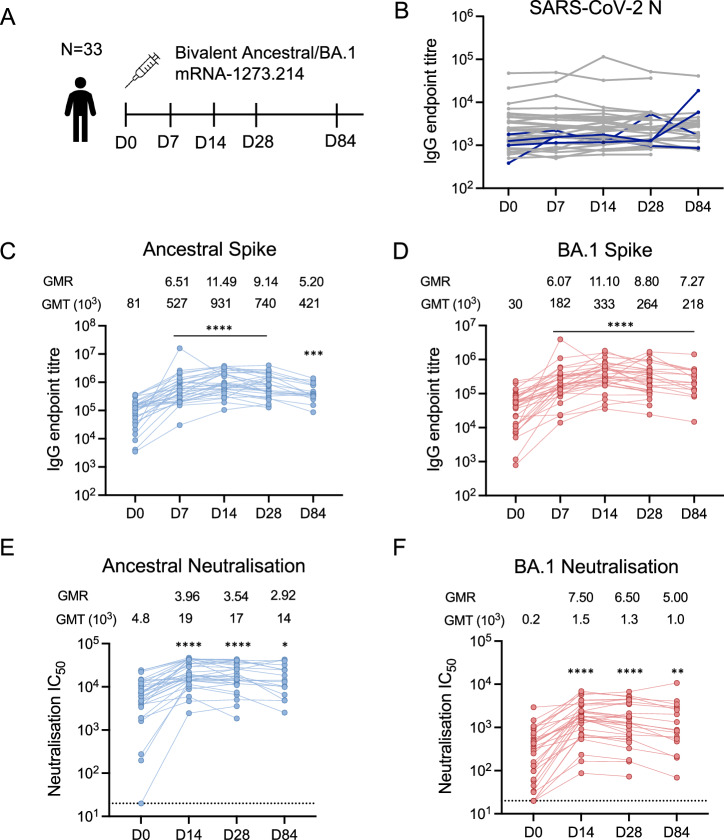
Antibody responses after bivalent COVID-19 booster vaccination. **A** Study design: healthy adults (*n* = 33) were administered a bivalent mRNA vaccine. Blood samples were collected pre-vaccination day (D)-0, and at days 7, 14, 28 and 84 post-vaccination. **B** IgG antibody endpoint titres against SARS-CoV-2 Nucleocapsid (N) protein. Individuals with a >4-fold change in titre are indicated in blue. **C** IgG antibody endpoint titres against Ancestral and **D** Omicron BA.1 spikes. **E** Neutralisation titres calculated by 50% virus inhibitory concentration (IC_50_) against Ancestral and **F** Omicron BA.1 live viruses. Geometric mean titres (GMT) and geometric mean ratios (GMR) are shown above each graph. For **C**–**F**, *n* = 32 at D0, D7, and D14; *n* = 31 at D28 and *n* = 17 at day 84. Asterisk indicates *p* value of Kruskal–Wallis test with Dunn’s post-test comparing D0 with D7, 14, 28 or 84. *p < 0.05, **p < 0.01, ***p < 0.001, ****p < 0.0001.

Thirty-three healthy adults (mean age 44.3 years, 16 female/17 male) were administered a booster dose of a bivalent ancestral/BA.1 COVID-19 mRNA vaccine (Fig. 1A, Supplementary Table 1). Antibodies against SARS-CoV-2 nucleocapsid (N) protein were monitored throughout the study to detect subclinical infections not reported by participants. All participants had detectable N-specific antibodies at baseline (Fig. 1B), despite one-third of them (10/33) having no self-reported prior infection. Four participants (depicted as blue lines) had >4-fold rise in N-specific antibodies between consecutive visits, suggesting a breakthrough infection. Consequently, the results of these participants at the impacted time points were excluded from further analysis.

### Rapid recall of plasma antibody responses after bivalent booster vaccination

All participants exhibited baseline binding IgG titres against both ancestral and BA.1 spike. IgG titres increased by day 7, peaked at day 14 and remained significantly elevated compared to baseline at day 84 (*p* < 0.001; Fig. 1C, D). While geometric mean titres (GMTs) were higher against ancestral than BA.1 spike at all time points, the geometric mean ratios (GMRs) between pre- and post-vaccination time points were similar across the two vaccine antigens (GMR day 14 ancestral = 11.49 vs. BA.1 = 11.10; Fig. 1C, D).

Virus neutralising antibodies (nAbs) are a correlate of protection against symptomatic SARS-CoV-2 infection^4^. We evaluated nAbs against the two vaccine strains using a live virus microneutralisation assay. The bivalent vaccine increased nAb titres against both viruses at day 14 (Fig. 1E, F), with titres maintained above baseline levels over the 84 days of follow-up (*p* = 0.012 for ancestral, *p* = 0.002 for BA.1). While higher nAb GMTs against ancestral virus were observed compared to BA.1 at all time points, GMRs were higher for BA.1 (GMR day 14 BA.1 = 7.19 vs. ancestral = 3.96).

### Baseline antigen-specific CD4 T cell frequencies correlate with post-vaccination nAb titres

**Fig. 2:**
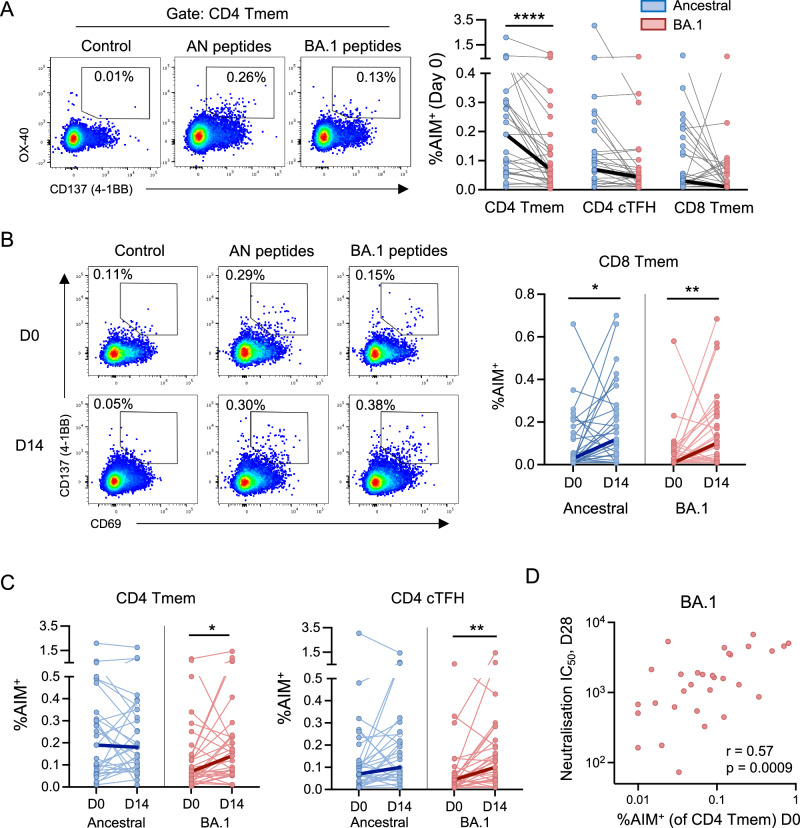
Spike-specific activation-induced marker (AIM) T-cell responses after bivalent COVID-19 booster vaccination. **A** Representative staining and frequencies of AIM^+^ CD4^+^ T memory (Tmem, non-naïve CXCR5^-^), CD4^+^ circulating T follicular helper (cTFH, CD45RA^−^CXCR5^+^) cells and CD8^+^ Tmem cells specific for the mutated epitopes on Omicron BA.1 spike and the corresponding epitopes in the Ancestral spike at baseline. AIM^+^ cells were defined as OX-40^+^CD137^+^ (for both CD4^+^ subsets) or CD69^+^CD137^+^ (for CD8^+^ T cells). **B** Representative staining and frequencies of CD8 Tmem recognising ancestral or BA.1 peptide at D0 and D14 post-vaccination. **C** Frequencies of CD4 Tmem and cTFH recognising ancestral or BA.1 peptides at D0 and D14 post-vaccination. **D** Correlation between frequencies of BA.1-specific AIM^+^ CD4^+^ Tmem at D0 and neutralisation titres against Omicron BA.1 at day 28 post-vaccination. *N* = 31 for all groups. Bold lines join the median values for each time point. Paired T-cell frequencies were analysed using the Wilcoxon signed-rank test. Correlation was performed by Spearman *r* test. *p < 0.05, **p < 0.01, ****p < 0.001.

In contrast to nAb responses, T cells induced by ancestral vaccination or infection are highly cross-reactive with variant spike proteins^5–7^. Nonetheless, a proportion of individuals exhibit comparatively poor recognition of the Omicron spike^8^. Here, we assessed T-cell responses to the bivalent vaccine using peptide pools covering the mutated regions in the Omicron BA.1 spike and the corresponding regions in the ancestral spike in an ex-vivo T-cell stimulation assay (cell subset definitions and gating shown in Supplementary Fig. 1A). At baseline, frequencies of AIM^+^ CD4^+^ T memory (Tmem) cells recognising the BA.1 mutated peptides (median 0.07%) were significantly lower than the frequencies of cells recognising the ancestral (AN) equivalent epitopes (median 0.19%) (2.7-fold difference, *p* < 0.0001; Fig. 2A). Differences were less pronounced among the AIM^+^ CD4^+^ circulating T follicular helper (cTFH) compartment (median 0.07% for AN vs 0.04% for BA.1), while peptide-specific CD8^+^ Tmem frequencies were low overall (0.03% for AN vs 0.01% for BA.1; Fig. 2A).

Vaccination drove a significant increase in CD8^+^ Tmem recognising both ancestral and BA.1 mutated peptides at day 14 (*p* = 0.013 for AN, *p* = 0.002 for BA.1; Fig. 2B). Sub-phenotyping based on memory subset revealed that Temra cells (CD45RA+ CCR7−) were the most abundant antigen-specific population, followed by Tem (CD45RA− CCR7−) and then Tcm (CD45RA− CCR7+) subsets, regardless of specificity or timepoint (Supplementary Fig. 1B). Compared to baseline, spike-specific Temra cells were significantly increased at day 14 for both AN and BA.1 (*p* = 0.013 and 0.001, respectively). Frequencies of antigen-specific CD4^+^ T cells recognising the ancestral-derived peptides were relatively stable following vaccination (Fig. 2C). In contrast, frequencies of both Tmem and cTFH specific to the BA.1 mutated peptides increased following vaccination (2-fold change for Tmem, *p* = 0.016, 2.3-fold change for cTFH, *p* = 0.001; Fig. 2C). Spike-specific CD4 Tmem exhibited a combination of Tcm and Tem phenotypes at both timepoints and regardless of AN or BA.1 specificity (Supplementary Fig. 1C). The increase in BA.1-specific Tmem at day 14 was attributed to a significant increase in the frequency of Tcm (*p* = 0.004), although there was also a similar trend toward increased frequencies of Tem (*p* = 0.061). Collectively, bivalent vaccination boosted both CD4 and CD8 T cell responses toward novel epitopes present in the BA.1 spike protein.

Previous studies have suggested that pre-vaccination CD4 T cell memory can predict post-vaccination antibody responses^9^. We, therefore, assessed whether spike-specific T-cell responses can predict nAb titres following bivalent vaccination. Interestingly, frequencies of BA.1-specific CD4^+^ Tmem at baseline correlated with BA.1 nAb titres at 28 days post-vaccination (Spearman *r* = 0.57, *p* = 0.0008; Fig. 2D), suggesting a role for T cell help and/or prior exposure to BA.1 in determining post-booster neutralising titres.

### Bivalent booster vaccination predominantly drives activation and expansion of memory B cells cross-recognising ancestral and BA.1 spike

**Fig. 3:**
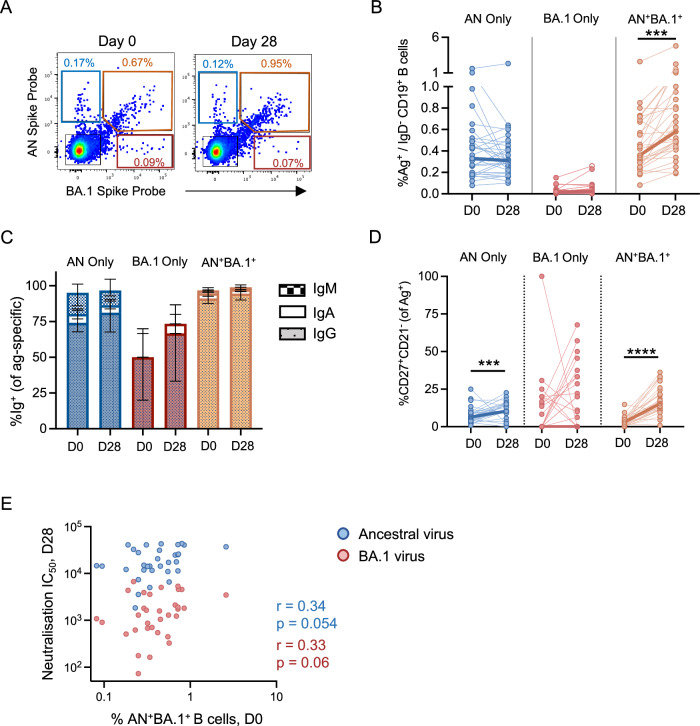
Spike-specific B-cell responses after bivalent COVID-19 booster vaccination. **A** Representative staining of antigen-specific memory B cells (CD19^+^IgD^−^) at baseline and 28 days post-vaccination. Two fluorochrome-conjugated spike probes were used to differentiate B-cell populations that were either specific for Ancestral (AN only), Omicron BA.1 (BA.1 only) or both spike proteins (AN^+^BA.1^+^). **B** Frequencies of antigen (Ag)-specific IgD^−^ CD19^+^ B cells at days 0 and 28. **C** Proportion of IgG^+^ (solid bars), IgA^+^ (open bars), and IgM^+^ (checked bars) cells in AN, BA.1, or AN^+^BA.1^+^ MBC populations. **D** Proportion of activated CD27^+^ CD21^−^ cells among MBC populations. **E** Correlation between baseline frequencies of AN^+^BA.1^+^ B cells and neutralisation titres against ancestral and Omicron BA.1 viruses at D28. *N* = 31 for all groups. Bold lines join the medians for each group. B-cell frequencies were analysed by the Wilcoxon signed-rank test. Correlations were analysed by Spearman *r* test. ***p < 0.001, ****p < 0.0001.

Memory B cells provide a mechanism for rapid supplementation of protective immunity upon re-infection or vaccination via differentiation into antibody-secreting cells. We assessed spike-specific B cell (CD19+ IgD−) responses using flow cytometry and probes of fluorochrome-conjugated ancestral and Omicron BA.1 spike proteins (gating shown in Supplementary Fig. 2). Three B-cell populations could be resolved: those binding the ancestral spike (AN^+^ only), binding Omicron BA.1 spike (BA.1^+^ only) or those cross-reactive to both (AN^+^ BA.1^+^; Fig. 3A). Following bivalent vaccination, a significant increase in the frequencies of AN^+^ BA.1^+^ cross-reactive B cells was observed (*p* < 0.001), in contrast to the minimal change in frequency of AN^+^ only or BA.1^+^ mono-specific populations (Fig. 3B). Post-vaccination frequencies of BA.1^+^ cells were generally low in all individuals (median 0.023%), while most subjects had clear populations of AN^+^ (median 0.31%) or AN^+^ BA.1^+^ (median 0.58%) B cells.

We next investigated the distribution of immunoglobulin isotypes and the activation status of spike-specific B cells over the course of vaccination. In both pre- and post-vaccination, all spike antigen-specific MBC populations were predominantly IgG, with no substantial changes between day 0 and 28 (Fig. 3C). To identify activated B cells, we assessed the frequency of CD27^+^ memory B cells that downregulated expression of CD21. In response to bivalent booster immunisation, we observed increased proportions of CD27^+^CD21^-^ cells in all three spike-specific B cell populations, with the most consistent activation in AN^+^ BA.1^+^ cells (median 3.16% at day 0 vs 15.30% at day 28, *p* < 0.0001; Fig. 3D).

Finally, we sought to determine whether the magnitude of the baseline MBC pool was associated with the post-vaccine serological response. In contrast to the relationship between baseline CD4 T cell memory and nAb titres, there was only a weak association between pre-vaccination cross-reactive B cell frequencies (*p* = 0.054 for ancestral virus, *p* = 0.06 for BA.1; Fig. 3E). No correlation was found between nAb titre and the frequency of either AN^+^ only or BA.1^+^ only populations.

### Vaccine-induced antibodies and B cells cross-react to Omicron XBB subvariant

**Fig. 4:**
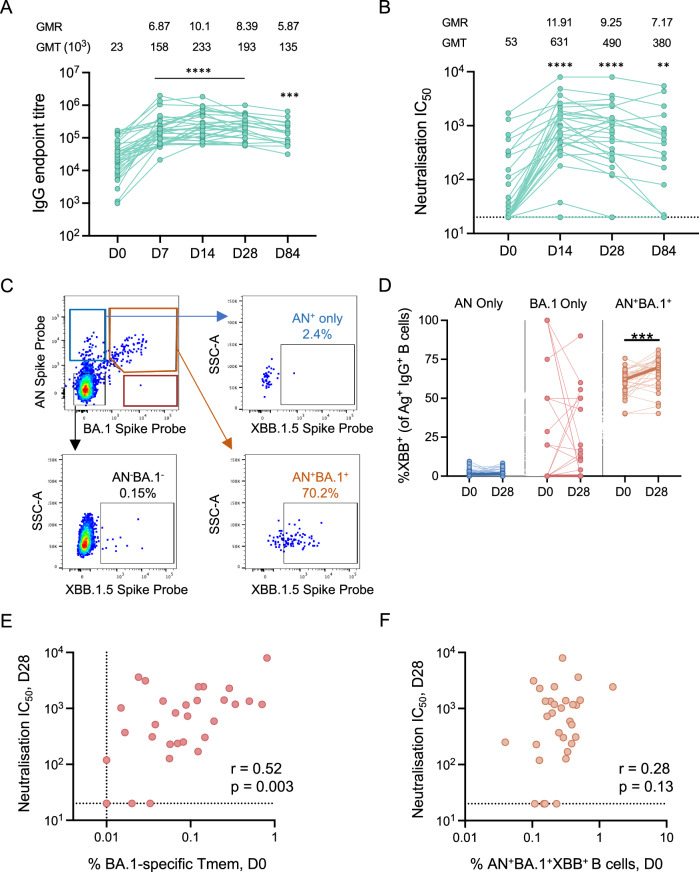
Cross-reactive antibody and B-cell responses to Omicron XBB after bivalent booster vaccination. **A** IgG endpoint titres and (**B**) live virus IC_50_ titres against XBB spike. The dotted line indicates the limit of detection. Geometric mean titres (GMT) and geometric mean ratios (GMR) are shown above the graph. Asterisk indicates *p* value of Kruskal–Wallis test with Dunn’s post-test comparing D0 with D7, 14, 28 or 84. **C** Representative staining of XBB.1.5 spike probe among populations of AN^+^, AN^+^BA.1^+^ or AN^-^BA.1^−^ IgG^+^ memory B cells. **D** Proportion of XBB^+^ cells in Ag^+^ IgG^+^ B cells at baseline and day 28 post-vaccination. Bold lines join the median for each time point. *N* = 32 at days 0 and 14; *N* = 31 at day 28 and *N* = 17 at day 84. B-cell proportions were analysed using a non-parametric paired-sample Wilcoxon signed-rank test. **E** Correlation between frequencies of BA.1-specific AIM^+^ CD4^+^ Tmem or **F** frequencies of AN^+^BA.1^+^XBB.1.5^+^ cross-reactive MBC at D0 and neutralisation titres against XBB at D28 post-vaccination (*n* = 31). Correlation assessed by Spearman *r* test. **p < 0.01, ***p < 0.001, ****p < 0.0001.

Shortly after the bivalent vaccine roll-out in Australia (September 2022), the previously circulating BA.1 variant was replaced with XBB (lineages XBB.1.16, XBB.1.5, and XBB.1.9), which circulated widely until late 2023. Hence, we assessed antibodies and B cells generated by the bivalent ancestral and Omicron BA.1 booster vaccine for cross-reactivity against XBB.1.5. We found an increase in IgG antibodies binding to the XBB.1.5 spike as early as 7 days after vaccination, with GMTs and GMRs peaking at 14 days post-vaccination (Fig. 4A). At baseline, 15/33 participants had detectible neutralising activity against live XBB virus (Fig. 4B). Vaccination elicited nAb in 91% of participants, with only 3 individuals failing to develop detectable XBB-specific nAb at either 14- or 28-days post-vaccination (Fig. 4B). XBB nAb titres remained significantly elevated at day 84 of follow-up compared to baseline (p = 0.0032; Fig. 4B), although at all timepoints titres against XBB remained markedly lower than against ancestral and BA.1 viruses (Figs. 4B and 1E, F).

To identify MBC populations with cross-reactivity to the XBB.1.5 spike, we examined the extent of XBB.1.5 probe binding within AN^+^ only, BA.1^+^ only, AN^+^ BA.1^+^ or AN^-^BA.1^-^ IgG^+^ B-cell populations (Fig. 4C). The vast majority of XBB.1.5 recognition occurred within the AN^+^ BA.1^+^ MBC compartment, with minimal cross-reactivity between XBB.1.5 and AN^+^ only cells, and sporadic XBB.1.5 binding within the minor BA.1^+^ only subset (Fig. 4D). Vaccination had a modest, but significant, impact on the proportion of XBB-binding cells within the AN^+^BA.1^+^ population (Fig. 4D; median 62.1% at day 0 versus 69.8% at day 28; *p* < 0.001).

Given the relationship between vaccine-strain nAb titres and CD4 T cell memory (Fig. 2D), we performed a similar correlation for XBB nAb titres at D28 with either baseline BA.1-specific CD4 Tmem (Fig. 4E) or baseline AN^+^BA.1^+^XBB^+^ MBC (Fig. 4F). Once again, baseline CD4 T cell memory correlated with post-vaccine nAb (*p* = 0.003, *r* = 0.52), while no such relationship was present for cross-reactive MBC (*p* = 0.13, *r* = 0.28).

### Predictors of vaccine-elicited neutralisation against antigenic variant viruses

Considering the broad cross-reactivity of spike-specific memory B cells, it is unsurprising that recipients of the bivalent mRNA vaccine showed expansion of serological responses able to recognise variant XBB spike. Nevertheless, the wide variation in serum XBB neutralising activity between participants suggests that booster immunisation may have provided differential protection against XBB viruses within vaccine recipients. To better understand the demographic and immunologic factors that influence post-vaccination neutralising titres, we constructed a multiple regression model. The model took into account participant age, gender, number of prior COVID-19 vaccinations, number of self-reported prior infections, days since last antigen exposure (either vaccination or infection), baseline IgG titres against Ancestral or BA.1 spike, baseline nAb titres against ancestral, BA.1 or XBB virus, baseline frequencies of Ancestral, BA.1 or cross-reactive memory B cells, and baseline frequencies of AIM^+^ CD4^+^ cTFH or Tmem.

Post-vaccination nAb titres against either ancestral or BA.1 virus were best predicted by a combination of baseline ancestral nAb titres and baseline ancestral spike-specific CD4 Tmem frequencies (Table 1). We next constructed a similar model to assess the variables that predict the post-vaccination nAb titre against XBB. Once again, baseline ancestral nAb titres and baseline spike-specific CD4 Tmem frequencies were found to be the only significant independent predictors of XBB nAbs (Table 1), suggesting that, in this cohort, a combination of pre-existing CD4 T cell help and prior antibody responses to the ancestral strain were the strongest predictors of post-vaccination antibody breadth against a future viral variant.

**Table 1:** Significant predictors of post-vaccination titres

Variable	Ancestral		BA.1		XBB	
	Coefficient	*p*-Value	Coefficient	*p*-Value	Coefficient	*p*-Value
Baseline ancestral nAb titres	0.051	0.00041	0.12	<0.00001	1.1	<0.00001
Baseline ancestral frequency of spike-specific CD4 Tmem	0.022	0.017	0.046	0.0063	0.61	<0.00001

Independent predictors of post-vaccination neutralising antibody titres for models were determined using forward and backwards regression (for Ancestral and XBB variants) and forward regression (for BA.1 variant). For backwards regression of BA.1 nAbs, two additional variables were marginally significant (Supplementary Table 2).

## Discussion

In line with previous reports^10,11^, we find the administration of ancestral/Omicron BA.1 mRNA vaccines as a booster in healthy adults reliably induced the recall of spike-specific memory T and B cell responses, and drove an increase in spike-specific serum antibody. As reported with Omicron breakthrough infections, we similarly observed that antibody responses against Omicron appeared to be elicited from the selective recall of cross-reactive memory B cell responses seeded by ancestral-encoding vaccines rather than arising from de novo responses^12–16^. Such cross-reactivity has been reported using both whole spike antigen probes as well as RBD probes^17^, likely explaining the ability to elicit cross-reactive neutralising antibodies. While we did see evidence that low frequencies of BA.1-specific memory B cells were present and became activated following immunisation, they remained a minor proportion of the MBC pool at both time points. Repeated exposure to omicron spike protein may not alter this bias, as nearly 70% of the cohort self-reported a prior SARS-CoV-2 infection during the period in which BA.1/2/5 viruses circulated in Australia, making the bivalent vaccine likely their second exposure to a BA.1-like spike. Regardless, the recall of cross-reactive MBCs efficiently boosted both binding and neutralising antibody titres to the vaccine antigens, demonstrating that Omicron mono-specific MBCs are not required to drive robust serum neutralising activity.

In contrast to nAb, CD4 and CD8 T cell recognition of Omicron variants is relatively well conserved, although a small fraction of individuals show low responses to Omicron relative to ancestral spike^8^. We find a moderate reduction in spike-specific CD4 Tmem frequencies when directly comparing BA.1 mutated peptides to their ancestral counterparts, but similar responses for CD8 T cells. Booster vaccination was particularly effective at increasing the frequency of BA.1 spike-specific CD4 and CD8 T cells, as measured by the AIM assay. In contrast, ancestral spike-specific CD4 Tmem frequencies were relatively stable, a phenomenon which has previously been observed in some bivalent booster cohorts^10^. This may reflect the stable maintenance of spike-specific CD4 T cell populations through continual antigen exposures via vaccination and infection.

We found the ancestral/BA.1 bivalent booster was surprisingly effective at eliciting immunity against XBB strains, despite significant additional spike mutations and consequent shift in antigenicity. While assay variation makes it difficult to specify the exact value of a protective neutralising titre, we have previously estimated that a 50% protective titre in our assay is approximately 1.0 × 10^2^ ^6^; in this context, 76% of the bivalent vaccine cohort exhibited XBB nAb titres that exceeded this value. Nevertheless, the XBB nAb GMT was only 380, and we observed variation over a 2-log range. In an increasingly complex immunological environment with rich infection and vaccination histories, the deconvolution of the immune mechanisms driving the development of broadly protective antibody responses remains challenging. Nonetheless, our analysis indicates that subjects with high baseline ancestral CD4 Tmem and neutralisation titres had a higher propensity to develop robust titres of XBB nAbs after vaccination. Interestingly, neither the number or nature of prior SARS-CoV-2 exposures nor the time since most recent exposure was predictive of post-vaccination XBB nAb titres, with the caveat that we cannot assess the impact of asymptomatic infections. These results suggest that any waning of serum neutralisation titres over the course of 4–15 months is insufficient to markedly impact the post-booster serological response when considered in the context of other variables. Instead, the independent association of both CD4 T cell and humoral immune memory with vaccine responsiveness emphasises the key role that recall of existing cross-reactive T and B cell responses will continue to play in programming serological breadth against SARS-CoV-2 variants.

## Methods

### Clinical trial

An open-labelled trial of the bivalent COVID-19 booster vaccine was approved by the local Ethics Committees to be conducted at the Royal Melbourne Hospital (Study number 2021/272) and the University of Melbourne (Approvals 13793 and 23497). The trial was registered with the Australian New Zealand Clinical Trials Registry (#12622000411741, https://www.anzctr.org.au), with the primary clinical endpoints reported in Lee et al.^18^. Healthy adults (18–65 years) who had received 2 or 3 doses of any COVID-19 vaccine at least 4 months prior to enrolment, were immunised with the booster vaccine intramuscularly. Exclusion criteria included COVID-19 infection within 4 months of recruitment, significant immunosuppressive illnesses, and severe adverse events to any previous COVID-19 vaccination. All participants provided written informed consent before inclusion. All associated activities were carried out in compliance with approved guidelines. Prior infection and vaccination history were obtained via questionnaire. Participants who completed day 84 of follow-up at the time of the study, regardless of trial arm, were included (*n* = 33).

Plasma was collected and stored at −80 °C until used in serological assays. PBMCs were isolated using Ficoll-Paque, cryopreserved with 10% DMSO in FCS, and stored in liquid nitrogen until used in T- and B-cell assays.

### Vaccine

The bivalent SARS-CoV-2 ancestral/Omicron BA.1 mRNA vaccine (mRNA-1273.214, “SpikeVax”, Moderna) was approved as a booster dose for adults in Australia by the Therapeutic Goods Administration on 29/08/2022. One dose (0.5 mL) contained 50 µg mRNA (25 µg mRNA each of ancestral Wuhan Hu-1 and Omicron BA.1 spike) embedded in lipid nanoparticles and was administered intramuscularly.

### SARS-CoV-2 proteins, viruses, and peptides

Recombinant SARS-CoV-2 nucleocapsid protein and spike proteins from ancestral, Omicron BA.1 and XBB.1.5 strains were produced and purified in-house (as in ref. ^19^).

Live ancestral SARS-CoV-2 (VIC01) virus was grown in Vero cells in serum-free DMEM with 1 µg/ml TPCK trypsin, while live Omicron BA.1 and XBB strains were grown in Calu3 cells in DMEM with 2% FCS. Virus propagation and titration were performed as described previously^16^, and mean values of the 50% infectious dose (ID_50_) were obtained for each virus.

Peptide pools of 83 peptides covering 37 mutations of the spike glycoprotein in the SARS-CoV-2 Omicron BA.1 variant and reference peptide pools of 82 peptides covering the respective homologous domains in the wild-type ancestral strain were obtained commercially (Miltenyi Biotec). Peptides were 15 amino acids in length with 11 amino acid overlap. Lyophilised peptide pools were reconstituted according to the manufacturer’s recommendations, aliquoted and stored at −20 °C until use.

### IgG ELISA

Binding immunoglobin (Ig)G antibodies to SARS-CoV-2 nucleocapsid and spike were measured in ELISA (as in ref. ^16^). Briefly, serially diluted plasma samples were added in duplicates into 96-well Maxisorp plates (Thermo Fisher), pre-coated with 2 µg/mL recombinant nucleocapsid or spike protein overnight and blocked with 1% FCS in PBS for two hours at room temperature. HRP-conjugated anti-human IgG detection antibodies (Sigma) were added at 1:20,000 dilution and incubated for one hour at room temperature. The plates were developed using TMB substrate (Sigma), and the reaction was stopped by sulphuric acid before reading at 450 nm using a FLUOstar Omega microplate reader (BMG Labtech). Endpoint titres were calculated as the reciprocal plasma dilution giving 2 times the background value using a fitted curve 4-parameter log regression in Prism v10.2.0 for Mac (GraphPad Software).

### Microneutralisation assay

Neutralising antibodies against live SARS-CoV-2 viruses were measured with viability dye readout as described previously^16^. Briefly, serially diluted heat-inactivated plasma samples were added in duplicates into 96-well flat-bottom plates and incubated with SARS-CoV-2 ancestral, Omicron BA.1, or XBB live viruses at a final concentration of 2× ID_50_ at 37 °C for 1 h before adding 30,000 freshly trypsinised HEK293T-ACE2-TMPRSS2 (HAT24) cells in DMEM with 5% FCS. After 46 h of incubation at 37 °C, 10 µl of alamarBlue™ Cell Viability Reagent (ThermoFisher) was added into each well. The plates were incubated at 37 °C for 1 h and stopped with 1% SDS. The reaction was read at excitation wavelength 560 nm and emission wavelength 590 nm on a FLUOstar Omega microplate reader (BMG Labtech). All live virus procedures were performed in a biosafety level-3 facility. The half-maximal inhibitory concentration (IC_50_) values were determined using a fitted curve 4-parameter non-linear regression in Prism v10.2.0 for Mac (GraphPad Software) with a constraint to a minimum of 0% and a maximum of 100% neutralisation.

### T-cell activation-induced marker (AIM) assay

SARS-CoV-2 spike-specific T cells were detected using flow cytometric assay after in vitro stimulation of PBMCs with spike peptide pools (as in ref. ^20^). Briefly, PBMCs were thawed and stimulated with peptide pools covering the mutated regions on Omicron BA.1 spike and the respective regions on ancestral Wuhan spike at a final concentration of 1 µg/ml in 96-well U-bottom plates. Unstimulated wells (cells only) were used as controls. After 22 h of incubation at 37 °C, the cells were washed and stained for viability with Aqua fluorescent reactive dye (Thermo Fisher), then surface stained with CD3 BUV805 (SK7), CD20 BV510 (2H7), CD45RA PE-Cy7 (HI100), (BD Biosciences), CD4 BV605 (RPA-T4), CD8 BV650 (RPA-T8), CD69 APC-Fire750 (FN50), CD134/OX-40 PerCP-Cy5.5 (Ber-ACT35), CD137/4-1BB BV421 (4B4-1), CD197/CCR7 Alexa647 (G043H7) (BioLegend), and CD185/CXCR5 PE (MU5UBEE) (Invitrogen). Cells were washed twice with PBS + 1% FCS, fixed with Cytofix (BD Biosciences), and acquired on an LSR Fortessa flow cytometer using FACS Diva software (BD Biosciences).

### B-cell flow cytometric assay

SARS-CoV-2 spike-specific B cells were detected using fluorochrome-conjugated spike probes in flow cytometric assay^16^. Biotinylated recombinant spike proteins derived from the ancestral Wuhan Hu-1 and the Omicron BA.1 and XBB.1.5 strains were conjugated to streptavidin-APC, PE (Invitrogen), or BB515 (BD Biosciences) fluorochromes, respectively. PBMCs were thawed and stained with Aqua fluorescent reactive dye (Thermo Fisher) for viability before surface staining with spike probes, CD3 BV510 (OKT3), CD8a BV510 (RPA-T8), CD10 BV510 (HI10a), CD14 BV510 (M5E2), CD16 BV510 (3G8), CD27 BV605 (O323) (BioLegend), streptavidin BV510, CD21 BUV737 (B-ly4), IgG BV786 (G18-145), IgD PE-Cy7 (IA6-2), IgM BUV395 (G20-127) (BD Biosciences), CD19 ECD (J3-119) (Beckman Coulter), and IgA VioBlue (IS11-8E10) (Miltenyi Biotec). Cells were washed twice with 1% FCS and fixed with 1% formaldehyde (Polysciences) before acquiring an LSR Fortessa flow cytometer using FACS Diva software (BD Biosciences).

### Statistical analyses

Visualisation of data and statistical analyses were done in Prism v10.2.0 for Mac (GraphPad Software). Longitudinal measurements of antibody titre after vaccination were compared to baseline data using Kruskal–Wallis test with Dunn’s post-test between Day 0 and Days 14, 28 or 84. T-cell frequencies and B-cell frequencies were assessed by the Wilcoxon paired-sample signed rank test. Spearman correlation was used to assess the relationship between log-transformed neutralising antibody titres and T-cell or B-cell frequencies. Values at or below the limit of detection (LOD) of the assay were assigned the LOD value for the purposes of statistical comparisons. P values ≤ 0.05 were considered statistically significant.

Multivariate linear regression was used to assess the relationship between post-vaccine nAb titres and pre-vaccination measurements. Variables for inclusion in the final model were selected by performing both forward and backward regression, considering the results of both. Variables that were tested in the model were: age, gender, number of prior COVID-19 vaccinations, number of self-reported prior infections, days since last antigen exposure (either vaccination or infection), baseline IgG titres against Ancestral and BA.1 spike, baseline nAb titres against ancestral, BA.1 and XBB.1.5 virus, baseline frequencies of Ancestral and BA.1-specific memory B cells, and baseline frequencies of AIM^+^ CD4^+^ cTFH and Tmem. The analyses were performed in R (v4.3.1) using the *lm*, *censReg* and *stepAIC* functions. *P*-values of <0.05 were considered significant. To ensure that only significant variables were selected into the final model we set a penalty parameter*, k*, of the *stepAIC* function. Forward regression models were built in the following steps: intercept only; baseline AN nAb titres; baseline AN nAb titres + baseline spike-specific CD4 Tmem. Backward regression models were built by removing the following variables in a stepwise fashion: (A) for post-vaccine WT nAb titres—BA.1 B cells, prior vaccination status, spike-specific cTFH, BA.1 nAb titres, AN B cells, gender, prior infection status, age, days since last exposure, AN IgG, BA.1 IgG; (B) for post-vaccine BA.1 nAb titres—AN IgG, spike-specific cTFH, age, BA.1 nAb titres, gender, AN B cells, BA.1 IgG, prior vaccination status, prior infection status, BA.1 B cells; (C) for post-vaccine XBB nAb titres—spike-specific cTFH, prior vaccination status, gender, age, AN IgG, BA.1 B cells, AN B cells, BA.1 IgG, BA.1 nAb titres, prior infection status, days since last exposure.

## Supplementary information

Supplementary Material

## Data Availability

Data is provided within the manuscript or supplementary information files. All datasets for the study are available from the corresponding author upon reasonable request.
