# Supplementary material for: Bivalent mRNA booster vaccination recalls cellular and antibody immunity against antigenically divergent SARS-CoV-2 spike antigens
Source: NPJ Vaccines. 2025 Apr 18;10:74. doi: 10.1038/s41541-025-01129-6 (PMC12008365; doi:10.1038/s41541-025-01129-6)
Supplement: Supplementary file 1 — Supplementary Material [file 41541_2025_1129_MOESM1_ESM.pdf]

Supplementary Material

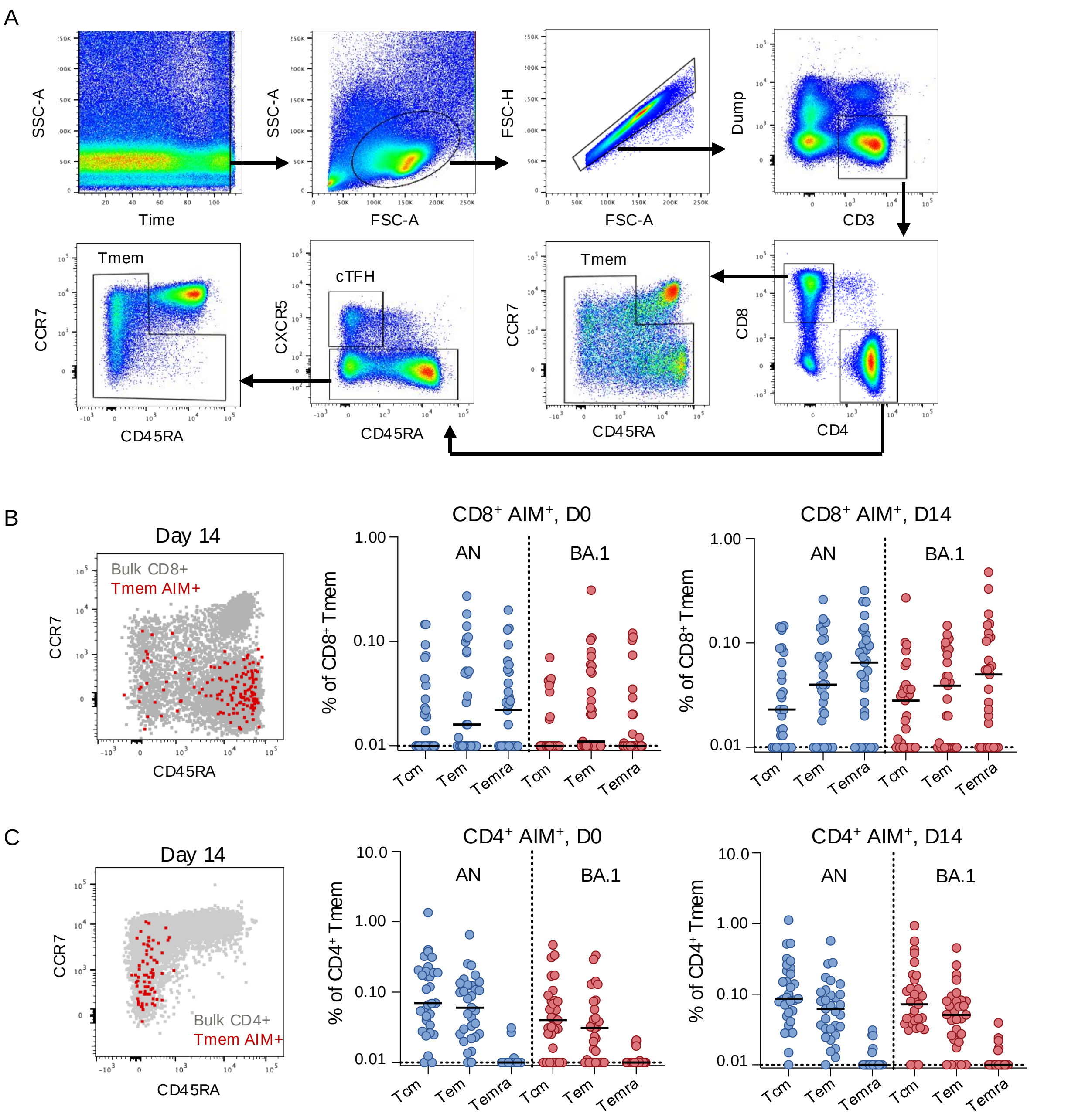

Supplementary Figure 1: Analysis of antigen-specific T cells.
(A) Gating strategy for antigen-specific T cells. Following stimulation, PBMC were gated based on time, lymphocytes identified by FSC and SSC, and singlets gated through FSC-A vs FSC-H. T cells were defined as $CD3^{+}Dump^{-}$ (live/dead and CD20), and either $CD8^{+}$ or $CD4^{+}$. Circulating TFH (cTFH) were defined as $CXCR5^{+}CD45RA^{-}$. Tmem were defined as either $CCR7^{+}CD45RA^{lo}$ or $CCR7^{-}$. (B) Frequency of $CD8^{+}$ AIM^{+} cells with a Tcm, Tem or Temra phenotype at baseline and day 14 post-vaccination. Tcm is defined as $CCR7^{+}CD45RA^{-}$, Tem is defined as $CCR7^{-}CD45RA^{-}$ and Temra is defined as $CCR7^{-}CD45RA^{+}$. FACS plot shows AIM^{+} cells ($CD69^{+}CD137^{+}$; red) overlaid on the bulk $CD8^{+}$ T cell population (grey). (C) Frequency of $CD4^{+}$ AIM^{+} cells with a Tcm, Tem or Temra phenotype at baseline and day 14 post-vaccination. FACS plot shows AIM^{+} cells ($OX\text{-}40^{+}CD137^{+}$; red) overlaid on the bulk CD4+ T cell population (grey). Lines indicate median.

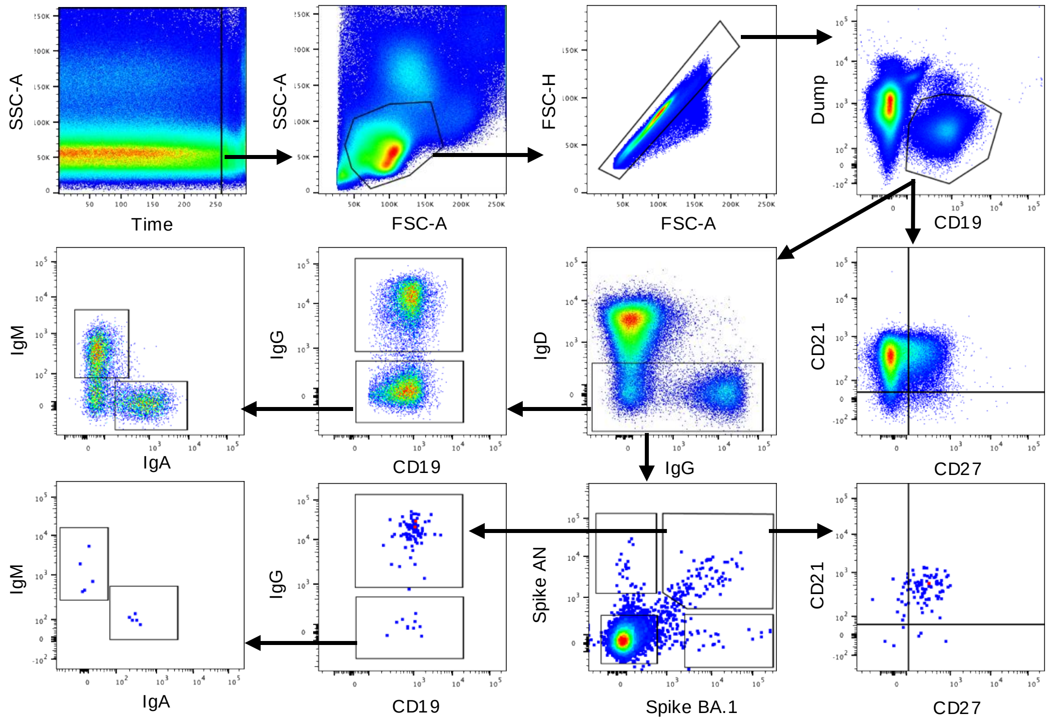

Supplementary Figure 2: Gating strategy for antigen-specific B cells.
For detection of antigen-specific B cells, PBMC were gated based on time, lymphocytes identified by FSC and SSC, and singlets gated through FSC-A vs FSC-H. B cells were defined as $CD19^{+}Dump^{-}$. Gates for activated ($CD27^{+}CD21^{-}$) cells and IgG, IgA and IgM isotypes were set based on total B cell or IgD^{-} B cell populations, respectively. Gates were then applied to each spike $probe^{+}$ IgD^{-} B cell subset.

Supplementary Table 1. Cohort demographics

Characteristics	n	%
Age		
25-34	11	33.3
35-44	6	18.2
45-54	3	9.1
55-62	13	39.4
Sex at birth		
Female	16	48.5
Male	17	51.5
COVID-19 Vaccination		
2 prior doses	2	6.1
3 prior doses	31	93.9
Prior COVID-19 infection (self reported)		
Yes	23	69.7
No	10	30.3

Supplementary Table 2: Significant predictors of post vaccination neutralising antibody titres using forwards and backwards regression
Estimated independent predictors of post vaccination neutralising antibody titres for models determined using forwards and backwards regression.

	Forwards Regression		Backwards Regression	
	Coefficient	p-value	Coefficient	p-value
**Ancestral**				
Baseline WT nAb titres	0.051	0.00041	0.051	0.00041
Baseline WT % spike-specific CD4 Tmem	0.022	0.017	0.022	0.017
**BA.1**				
Baseline WT nAb titres	0.12	<0.00001	0.1	<0.00001
Baseline WT % spike-specific CD4 Tmem	0.046	0.0063	0.047	0.003
Prior infection status	NA	NA	0.053	0.023
Days since last exposure	NA	NA	0.00022	0.022
**XBB**				
Baseline WT nAb titres	1.1	<0.00001	0.051	0.00041
Baseline WT % spike-specific CD4 Tmem	0.61	<0.00001	0.022	0.017
